# Comprehensive study of altered proteomic landscape in proximal renal tubular epithelial cells in response to calcium oxalate monohydrate crystals

**DOI:** 10.1186/s12894-020-00709-z

**Published:** 2020-08-31

**Authors:** Zhu Wang, Ming-xing Li, Chang-zhi Xu, Ying Zhang, Qiong Deng, Rui Sun, Qi-yi Hu, Sheng-ping Zhang, Jian-wen Zhang, Hui Liang

**Affiliations:** 1 Department of Urology, People’s Hospital of Longhua, Southern Medical University, Shenzhen, 518109 Guangdong China; 2 Laboratory of Molecular Pharmacology, Department of Pharmacology, School of Pharmacy, Southwest Medical University, Luzhou, 646000 Sichuan China; 3 Department of Laboratory Medicine, Third Affiliated Hospital of Sun Yat-sen University, Guangzhou, Guangdong 510630 People’s Republic of China

**Keywords:** Calcium oxalate crystal, Kidney stone, Protein expression profile, Renal epithelial cells

## Abstract

**Background:**

Calcium oxalate monohydrate (COM), the major crystalline composition of most kidney stones, induces inflammatory infiltration and injures in renal tubular cells. However, the mechanism of COM-induced toxic effects in renal tubular cells remain ambiguous. The present study aimed to investigate the potential changes in proteomic landscape of proximal renal tubular cells in response to the stimulation of COM crystals.

**Methods:**

Clinical kidney stone samples were collected and characterized by a stone component analyzer. Three COM-enriched samples were applied to treat human proximal tubular epithelial cells HK-2. The proteomic landscape of COM-crystal treated HK-2 cells was screened by TMT-labeled quantitative proteomics analysis. The differentially expressed proteins (DEPs) were identified by pair-wise analysis. Gene Ontology (GO) analysis and Kyoto Encyclopedia of Genes and Genomes (KEGG) pathway analysis of DEPs were performed. Protein interaction networks were identified by STRING database.

**Results:**

The data of TMT-labeled quantitative proteomic analysis showed that a total of 1141 proteins were differentially expressed in HK-2 cells, of which 699 were up-regulated and 442 were down-regulated. Functional characterization by KEGG, along with GO enrichments, suggests that the DEPs are mainly involved in cellular components and cellular processes, including regulation of actin cytoskeleton, tight junction and focal adhesion. 3 high-degree hub nodes, CFL1, ACTN and MYH9 were identified by STRING analysis.

**Conclusion:**

These results suggested that calcium oxalate crystal has a significant effect on protein expression profile in human proximal renal tubular epithelial cells.

## Background

Kidney stone is a common disease with prevalence and incidence increasing across the world that seriously affects human health. Generally, it can cause urinary retention, renal pelvis and ureteral hydrops, ureteral dilatation, renal function damage, and infection. The prevalence of kidney stones exhibited a significant increasing trend in the past decades in the mainland of China [1, 2]. The latest research shows that the prevalence of Chinese adult kidney stones is 5.8%, and the life-time prevalence in South China is as high as 26.6% [3]. Kidney stone disease mainly affects people aged 30–50 years old, and half the patients suffered kidney stone recurrence [4]. Kidney stone seriously affects the health and quality of life of patients, and has become a public health problem across the world.

Calcium Oxalate Monohydrate (COM) accounts for over 80% of the incidence of stones [5]. COM caused renal tubular epithelial cells expressing a variety of macromolecules [6], thus affecting adhesion, aggregation and growth of calcium oxalate crystals plays an important role in the formation of kidney stones [6, 7]. Studies reported that the COM crystal-cell interaction stimulates the expression of NADPH oxidase in renal tubular epithelial cells, triggers the massive production of reactive oxygen species, activates the nuclear factor-κB signal transduction pathway, and releases a large number of inflammatory factors and activates the inflammatory cascade, links to local intrarenal inflammation and kidney injury [8–11].

Recently, the mechanism of kidney stone formation has been increasingly concerned. Serval studies demonstrated that kidney stones are initiated by COM crystals deposition due to supersaturation of urinary calcium and oxalate ions [12]. These deposits may act as nidus for stone growth, adhere to apical surface of renal tubular epithelial cells via several crystal-binding molecules or potential crystal receptors [13]. Calcium oxalate stone matrix has been reported to include a large number of protein molecules, which were identified and evidenced to promote the aggregation, nucleation and growth of calcium oxalate crystals in kidney, which ultimately leads to the formation of stones [6, 7, 14–16]. CD44, OPN, MCP-1 and HA are the most widely studied proteins which calcium oxalate crystal attachment depends upon [8, 17]. However, various theories of pathogenesis of human kidney stones suggest that calcium oxalate stone formation is a multistep process which is too complex for simple understanding, a large number of protein molecules involved are still unknown and uninvestigated.

Crystal-cell interaction model is widely used for kidney stone research for better understanding of the pathogenic mechanisms of kidney stone formation [18]. MDCK renal tubular cells (a cell line derived from dog kidney exhibiting distal renal tubule phenotype) and HK-2 cells (an immortalized human kidney proximal tubule epithelial cell line) are the most frequently used cells in crystal-cell interaction model [18]. Although several previous studies have found some new candidate proteins, further studies are necessary, since the COM-crystals prepared by calcium chloride dihydrate and sodium oxalate are different from the clinical COM-stone samples and use of cells from different species of the nephron may result in different findings. We used HK-2 cells for it was derived from adult human kidney proximal tubule which is the major site of renal oxalate handling [19], represents a major potential advantage over currently available animal or human embryonic derived cell lines [20].

In this study, we aim to use TMT (Tandem Mass Tag)–based quantitative proteomics analysis to investigate the effects of calcium oxalate crystal on the differential protein expression profiles of human renal tubular epithelial cell HK-2, screen differentially expressed protein molecules and initially explore their functional roles in calcium oxalate stone formation and its resulting renal damage.

## Methods

### Cell culture

The immortalized proximal tubule epithelial cell line HK-2 (human kidney-2) was purchased from Bogoo Biotechnology.Co., Ltd. (Cat. BG005, Shanghai, China), and cultured with DMEM medium supplemented with 10% fetal bovine serum, 100 units/ml penicillin sodium, and 100 μg/ml streptomycin as described previously [14]. Cultures were maintained at 37 °C with 5% CO2 and saturated humidity [21–23].

### COM crystal preparation

The calcium oxalate kidney stone specimen used in this study was obtained from the Department of Urology, People’s Hospital of Longhua Shenzhen in 2018. After characterization by a stone component analyzer (LIIR-20, Lambda scientific, Tianjin China), the calcium oxalate kidney stone was fully crushed into powder by sterilized mortar and pestle and then prepared into COM suspension. Briefly, the crystals were suspended with serum-free DMEM at a final concentration of 100 μg/mL (per volume of medium) [14, 24], which was demonstrated not to cause severe cytotoxicity to renal tubular cells or increase percentage of cell death [24], but induce alterations in cellular proteome and reflect response of the renal epithelial cells to the COM crystals in vivo [18, 24, 25]. The kidney stone specimen was used in accordance with the hospital ethical review and the patient’s informed consent.

### Protein extraction and quality analyzation

HK-2 cells were seeded into 6-well plates at a density of 1 × 10^5^ cells per well and divided into two groups (*n* = 3 per group) when the cell density reached 70–80% confluence. The culture medium was replaced by either COM-crystal containing medium (with 100 μg/mL COM crystals) or COM-free medium. The cells were further maintained for 24 h. Cell pictures were taken after incubation by a microscope (GMSP-5, Shanghai Guangmi instrument Co.,ltd). Cell samples were harvested by cell scraping, repeatedly frozen and thawed, and then sonicated on ice for 2 min; After 4 °C, 12000 g centrifugation for 20 min, protein supernatant was collected for BCA quantification and SDS-PAGE electrophoresis.

### TMT-based quantitative proteomic analysis

TMT (Tandem Mass Tag) technology is a peptide in vitro labeling technology developed by Thermo Scientific, USA. The technology uses 10 isotopic labels to label the amino group of the peptide. After LC-MS/MS analysis, peak identification was performed to obtain a peak list and a reference database was established to identify peptides and proteins. Samples were analyzed by Shanghai Majorbio bio-pharm Biotechnology Co. Ltd. (Shanghai, China). The differential expressed proteins were screened based on the significance of *p* values, and the differentially expressed proteins were subjected to bioinformatics.

### GO and KEGG enrichment analysis

GO (Gene Ontology, http://www.geneontology.ory/) is a comprehensive database of gene-related research results from all over the world. The significant functional enrichment analysis of the differential proteins can explain the functional enrichment of the differential proteins and clarify the differences in the sample components at the functional level. This study used Goatools for enrichment analysis using Fisher’s exact test. KEGG (Kyoto Encyclopedia of Genes and Genomes) is a database resource for understanding high-level functions and utilities of the biological system, so we used KOBAS software to test the statistical enrichment of differential expression proteins in KEGG pathways.

### Differential protein interaction network analysis

The Search Tool for the Retrieval of Interacting Genes (STRING) database (http://string-db.org/), a database that provides experimental and predicted proteins interaction information [26], was used to build protein-protein interaction networks. In addition to mining data including experimental data and various databases, comprehensive scoring (0.4–1) is performed from the aspects of chromosome proximity, gene fusion, phylogenetics and gene-based co-expression based on chip data, thereby predicting the inter-protein interactions. Each node in the network represents a protein molecule, and the line represents the interaction between proteins. The wider the line, the higher the score, and the narrower the line, the lower the score. In this study, the cutoff confidence score of protein-interactions was ≥0.4.

### Statistics

Statistical analysis was performed using SPSS17.0 software. Quantitative data were analyzed by one-way ANOVA. Those with irregular variance were analyzed by rank sum test. Differences between samples were analyzed by LSD method, and the results were expressed as mean ± standard deviation. The difference was statistically significant at *p* < 0.05.

## Results

### Preparation of kidney stone treated HK-2 cells and quality control of TMT mass spectrometry samples

**Fig. 1:**
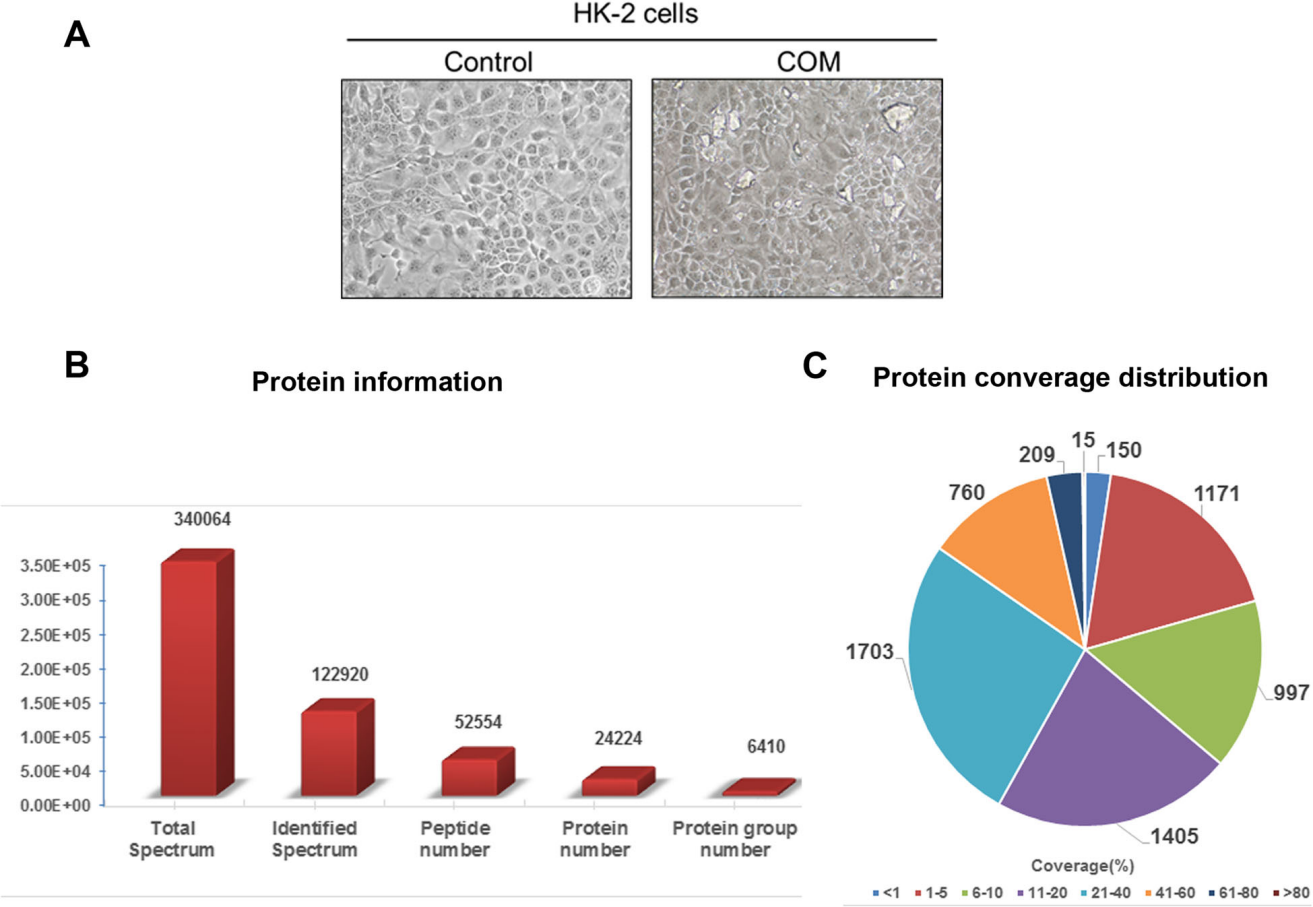
COM-HK-2 cell preparation and protein sample quality control. **a**. Representative pictures of calcium oxalate monohydrate crystals treated HK-2 cells and its’ parental control. COM crystal adhesion and cell-crystal interactions. Images were taken using phase-contrast microscopy. **b**. Protein information of quality control. **c**. Protein coverage distribution of quality control

The human proximal tubular epithelial cell HK-2 cells were suspended into single cells, seeded onto 6-well plates overnight. Crystal suspension of calcium oxalate stone with a final concentration of 100 μg/ml was added and incubated for 24 h. The calcium oxalate crystals adhered to the surface of HK-2 cells was clearly observed by phase-contrast microscopy (Fig. 1a). These COM crystals adhere tightly to the surface of the cells, which even cannot be removed by several washes with PBS. After incubation, cells were harvested by scraping, and prepared for quality control prior to TMT analysis. Quality control results showed that 24,224 proteins were identified in 122,920 identified spectrum (Fig. 1b). Protein coverage distribution analysis showed that 4137 proteins were covered over 10% (Fig. 1c), suggesting that the sample preparation was successful and could be proceeded for TMT analyzation.

### Proteome alterations of HK-2 cells after adhered by calcium oxalate crystals

**Fig. 2:**
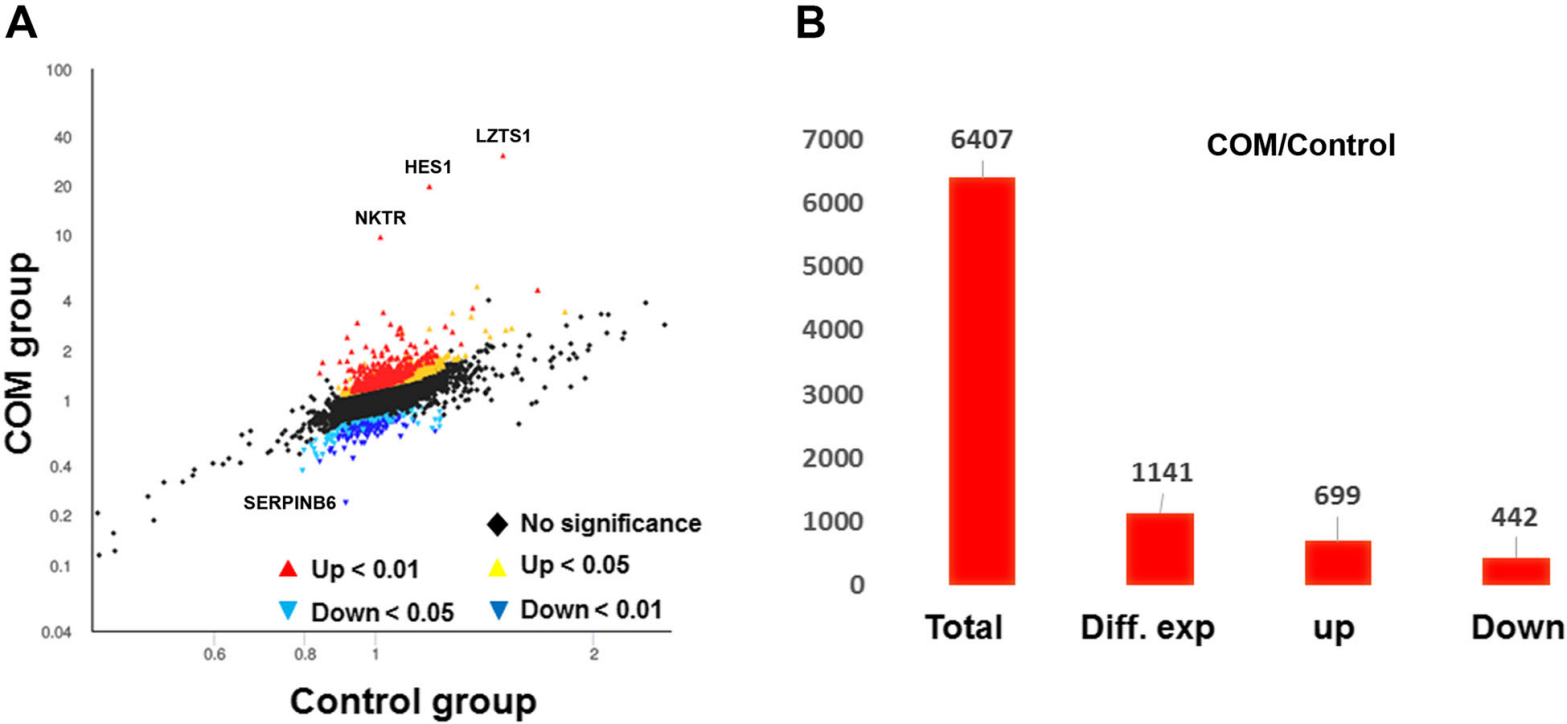
Proteome alterations of HK-2 cells after treated with calcium oxalate crystals. **a**. Scatter plot of differential expressed proteins statistics. **b**. Histogram of differential expressed proteins

The screening criteria for significant differentially expressed proteins (DEPs) were *p* < 0.05 and *FC* < 0.83 or *FC* > 1.20. The scatter plot of the DEPs is shown in Fig. 2a. Results showed that among the total number of 6407 target proteins, 1141 (17.8%) proteins changed significantly, of which 699 (61.3%) proteins were up-regulated (Fig. 2b), 442 (38.7%) proteins were down-regulated expression. The proteins with *p* > 0.05 and *FC* > 2 (Table 1) or *FC* < 0.6 (Table 2) were selected for further bioinformatics analyzation.

**Table 1:** Upregulated proteins in COM-stimulated HK-2 cells (*P* < 0.05, *FC* > 2)

Gene name	Accession	FC (C/H)	***P*** value (C/H)	Description
LZTS1	Q9Y250	20.20	0.001	Leucine zipper putative tumor suppressor 1 OS=Homo sapiens GN = LZTS1 PE = 1 SV = 3
HES1	Q14469	16.53	0.001	Transcription factor HES-1 OS=Homo sapiens GN=HES1 PE = 1 SV = 1
NKTR	P30414	9.53	0.001	NK-tumor recognition protein OS=Homo sapiens GN=NKTR PE = 1 SV = 2
KLK15	M0R2F7	3.51	0.033	Kallikrein-15 (Fragment) OS=Homo sapiens GN=KLK15 PE = 4 SV = 1
CLPS	P04118	3.30	0.002	Colipase OS=Homo sapiens GN=CLPS PE = 1 SV = 2
DPP8	Q6V1X1	3.09	0.004	Dipeptidyl peptidase 8 OS=Homo sapiens GN = DPP8 PE = 1 SV = 1
CEP128	Q6ZU80	2.77	0.004	Centrosomal protein of 128 kDa OS=Homo sapiens GN=CEP128 PE = 1 SV = 2
PHRF1	Q9P1Y6	2.74	0.003	PHD and RING finger domain-containing protein 1 OS=Homo sapiens GN=PHRF1 PE = 1 SV = 3
HNRNPD	D6RBQ9	2.64	0.007	Heterogeneous nuclear ribonucleoprotein D0 (Fragment) OS=Homo sapiens GN=HNRNPD PE = 1 SV = 1
ANXA2	H0YL33	2.63	0.015	Annexin (Fragment) OS=Homo sapiens GN = ANXA2 PE = 1 SV = 1
TMEM141	Q96I45	2.61	0.000	Transmembrane protein 141 OS=Homo sapiens GN = TMEM141 PE = 1 SV = 1
FAU	E9PM49	2.53	0.001	40S ribosomal protein S30 (Fragment) OS=Homo sapiens GN=FAU PE = 1 SV = 8
TMSB10	P63313	2.49	0.009	Thymosin beta-10 OS=Homo sapiens GN = TMSB10 PE = 1 SV = 2
CFL1	E9PP50	2.38	0.013	Cofilin-1 (Fragment) OS=Homo sapiens GN=CFL1 PE = 1 SV = 8
RPS29	P62273	2.35	0.024	40S ribosomal protein S29 OS=Homo sapiens GN = RPS29 PE = 1 SV = 2
ACTB	C9JTX5	2.34	0.000	Actin, cytoplasmic 1 (Fragment) OS=Homo sapiens GN = ACTB PE = 1 SV = 1
ZNF12	P17014	2.32	0.001	Zinc finger protein 12 OS=Homo sapiens GN = ZNF12 PE = 1 SV = 3
GCHFR	P30047	2.27	0.013	GTP cyclohydrolase 1 feedback regulatory protein OS=Homo sapiens GN = GCHFR PE = 1 SV = 3
ZNF146	Q15072	2.23	0.000	Zinc finger protein OZF OS=Homo sapiens GN = ZNF146 PE = 1 SV = 2
TMEM222	Q9H0R3	2.17	0.002	Transmembrane protein 222 OS=Homo sapiens GN = TMEM222 PE = 1 SV = 2
ZNF574	A0A0C4DFM2	2.16	0.000	HCG1643764, isoform CRA_b OS=Homo sapiens GN = ZNF574 PE = 1 SV = 1
TSHZ1	Q6ZSZ6	2.11	0.006	Teashirt homolog 1 OS=Homo sapiens GN = TSHZ1 PE = 1 SV = 2
ITGA5	P08648	2.04	0.007	Integrin alpha-5 OS=Homo sapiens GN=ITGA5 PE = 1 SV = 2
BTF3	D6RDG3	2.02	0.004	Transcription factor BTF3 (Fragment) OS=Homo sapiens GN=BTF3 PE = 1 SV = 3

**Table 2:** Down-regulated proteins in COM-stimulated HK-2 cells (*P* < 0.05, *FC* < 0.6)

Gene name	Accession	FC (C/H)	***P*** value (C/H)	Description
PRDX1	Q06830	0.57	0.001	Peroxiredoxin-1 OS=Homo sapiens GN=PRDX1 PE = 1 SV = 1
NME2P1	O60361	0.57	0.029	Putative nucleoside diphosphate kinase OS=Homo sapiens GN=NME2P1 PE = 5 SV = 1
SLC39A10	Q9ULF5	0.57	0.001	Zinc transporter ZIP10 OS=Homo sapiens GN=SLC39A10 PE = 1 SV = 2
SERPINB1	P30740	0.56	0.006	Leukocyte elastase inhibitor OS=Homo sapiens GN=SERPINB1 PE = 1 SV = 1
FAM133B	Q5BKY9	0.56	0.014	Protein FAM133B OS=Homo sapiens GN=FAM133B PE = 1 SV = 1
PRDX2	P32119	0.56	0.003	Peroxiredoxin-2 OS=Homo sapiens GN=PRDX2 PE = 1 SV = 5
MYL12A	J3QRS3	0.56	0.012	Myosin regulatory light chain 12A OS=Homo sapiens GN = MYL12A PE = 1 SV = 1
MYH14	Q7Z406	0.55	0.002	Myosin-14 OS=Homo sapiens GN = MYH14 PE = 1 SV = 2
MAP10	Q9P2G4	0.55	0.017	Microtubule-associated protein 10 OS=Homo sapiens GN = MAP10 PE = 1 SV = 2
PADI2	Q9Y2J8	0.54	0.012	Protein-arginine deiminase type-2 OS=Homo sapiens GN=PADI2 PE = 1 SV = 2
C14orf1	Q9UKR5	0.54	0.003	Probable ergosterol biosynthetic protein 28 OS=Homo sapiens GN=C14orf1 PE = 1 SV = 1
MYH9	P35579	0.53	0.010	Myosin-9 OS=Homo sapiens GN = MYH9 PE = 1 SV = 4
MT-ND2	P03891	0.50	0.010	NADH-ubiquinone oxidoreductase chain 2 OS=Homo sapiens GN = MT-ND2 PE = 1 SV = 2
APITD1-CORT	A0A087WT10	0.47	0.001	APITD1-CORT readthrough OS=Homo sapiens GN = APITD1-CORT PE = 1 SV = 1
TUFT1	Q9NNX1	0.47	0.015	Tuftelin OS=Homo sapiens GN = TUFT1 PE = 1 SV = 1
SERPINB6	A0A087X1N8	0.26	0.000	Serpin B6 OS=Homo sapiens GN=SERPINB6 PE = 1 SV = 1

### Cluster analysis of differentially expressed proteins

**Fig. 3:**
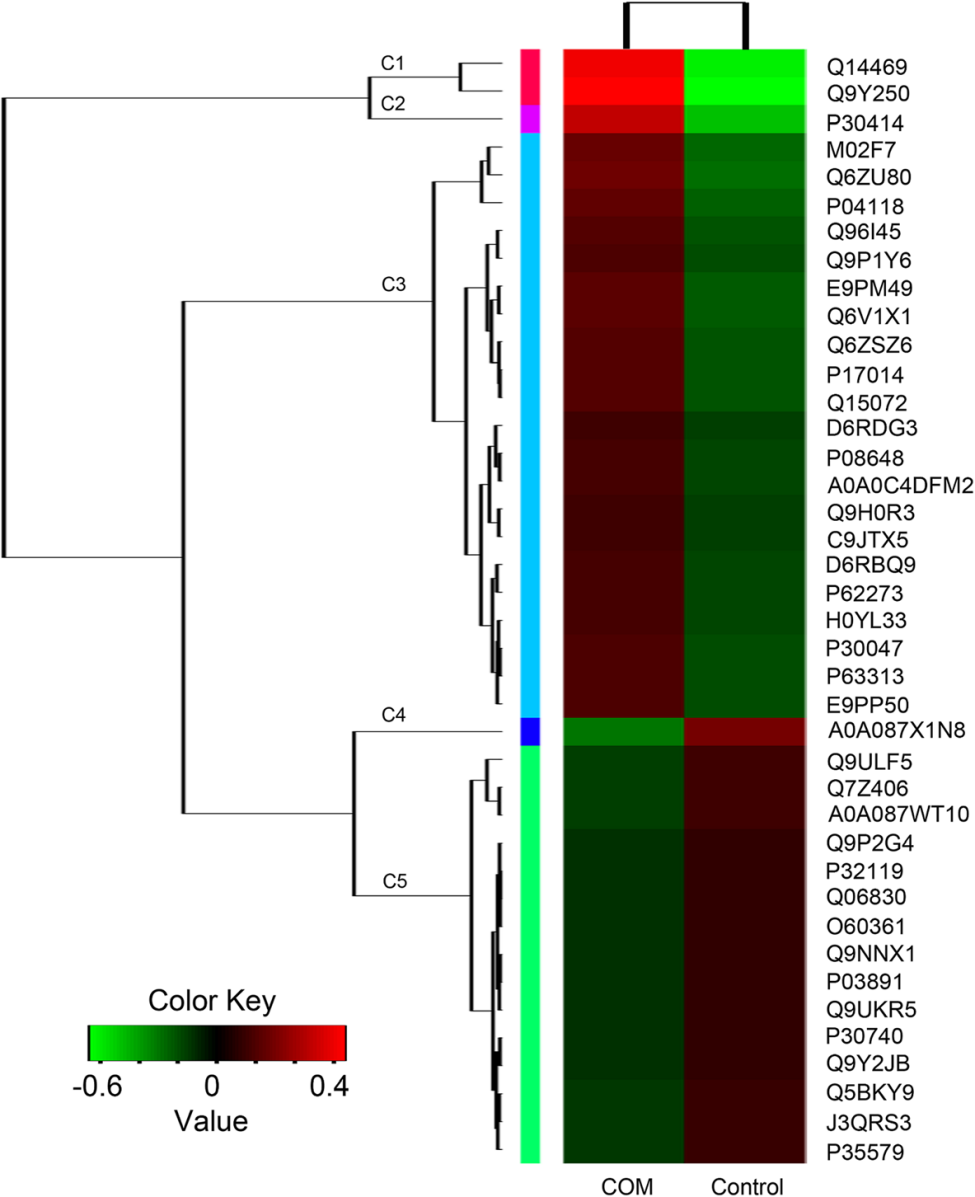
Heatmap and cluster analysis of differential expressed proteins. Proteins fold change (FC) > 2 or < 0.6 and *p* < 0.05 were enriched

Two-dimensional hierarchical clustering feature of the 40 selected DEPs was presented in a heatmap (Fig. 3). Columns in the heat map represents COM-treated group (*n* = 3) and Control group (*n* = 3) respectively. The color in the figure indicates the relative expression of the protein in the sample, the red color indicates that the protein is expressed higher in the sample, and the green color represents the lower expression level. The trend of the amount of expression is shown in the color code. On the left is a dendrogram of protein clustering. Cluster analysis showed that the selected DEPs can be divided into 5 clusters (C1 to C5) based on the heat map signal intensity. The cluster 1 (C1) included two proteins (LZTS1, HES1) which showed the highest expression level in the COM treated group compare to that in control group. The cluster 3 (C3) included 21 proteins which were higher expressed in COM treated group. By contrast, the cluster 5 (C5) consisted 15 proteins which were decreased in COM treated group.

### GO and KEEG enrichment analyses

**Fig. 4:**
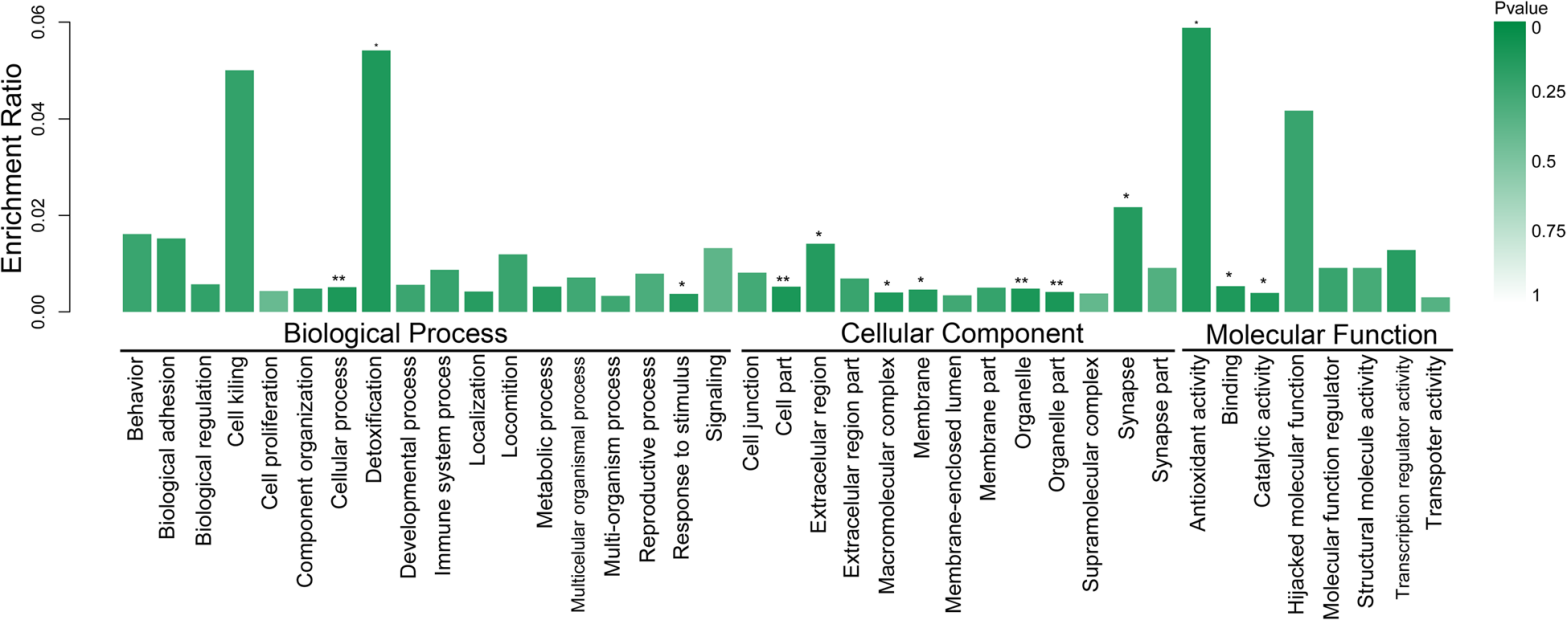
GO analysis. Each column is a GO term, the abscissa indicates the GO name and the classification, the height of the column indicates the enrichment rate, and the *p* value is represented by the color. The darker the color, the more significant. ***, *p* < 0.001; **, *p* < 0.01; *, *p* < 0.05

**Fig. 5:**
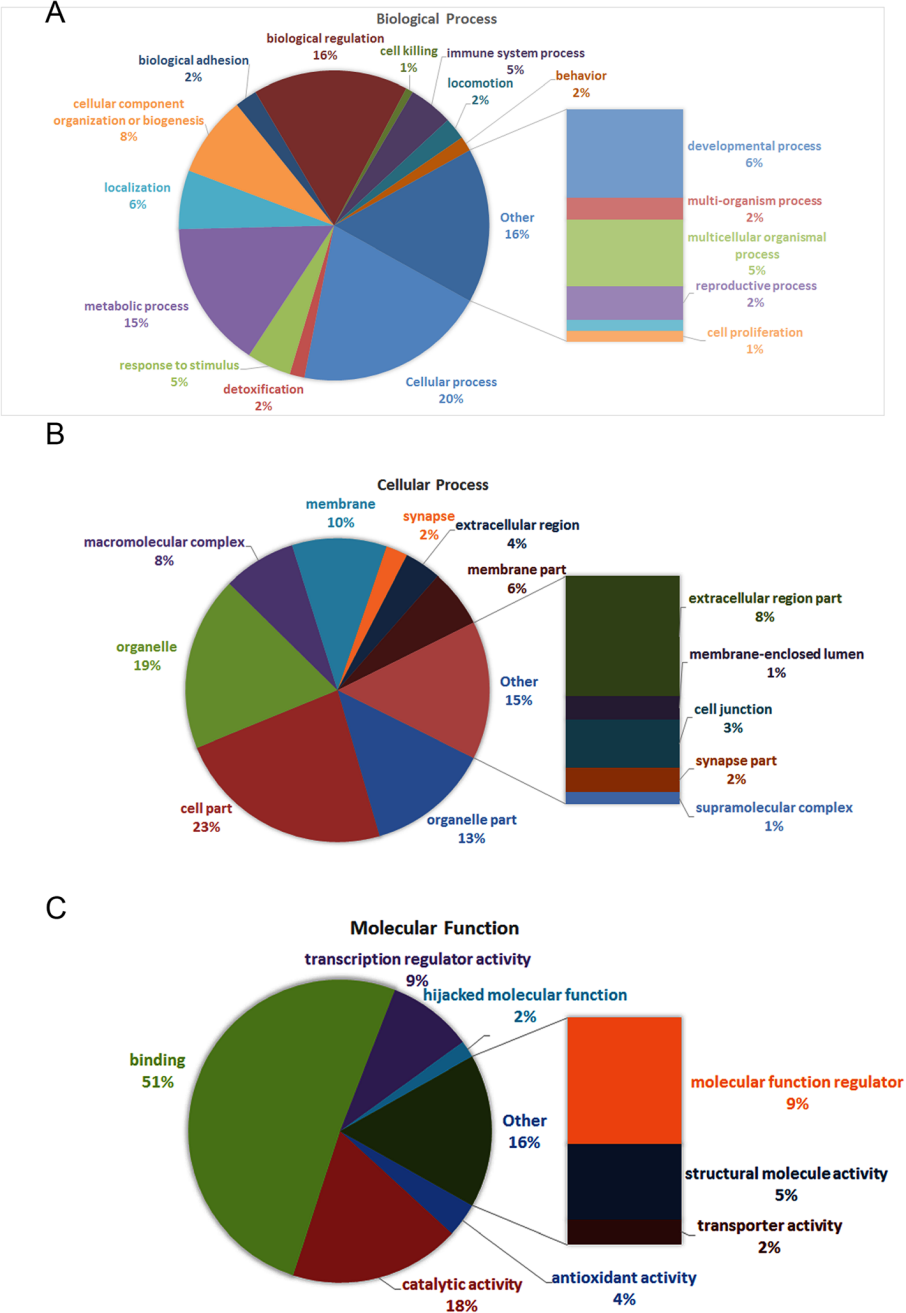
GO level-2 distribution of differential expression proteins. **a**. Biological process. **b**. Cellular process. **c**. Molecular function. Results showed that most of the differential expressed proteins are related to cellular process, cell part and binding

The significant functional enrichment analysis of GO proteins on differential proteins can explain the functional enrichment of differential proteins and clarify the differences exhibited in the samples at the functional level. To investigate the biological roles of the DEPs altered by COM crystals in HK-2 cells, a categorized GO enrichment analysis was performed. According to the gene function classification of GO, the selected DEPs were mainly related to cellular process, cell part, organelles, binding and catalytic activity (Figs. 4 and 5). For the response to the COM crystals, cell part (*p* = 0.002) and cellular process (*p* = 0.009) were the most significantly enriched functional terms for cellular component (CC) and biological processes (BP), respectively. Most of the candidate proteins are involved in cellular component (CC) as showed in Table 3.

**Table 3:** GO terms of the differentially expressed proteins involved in COM-stimulated HK-2 cells

Name	Namespace	Count	*P* value
Cell part	Cellular component	30	0.002
Organelle	Cellular component	24	0.003
Membrane	Cellular component	13	0.038
Organelle part	Cellular component	17	0.001
Macromolecular complex	Cellular component	10	0.021
Extracellular region	Cellular component	5	0.046
Synapse	Cellular component	3	0.044
Cellular process	Biological process	26	0.009
Response to stimulus	Biological process	6	0.046
Detoxification	Biological process	2	0.020
Antioxidant activity	Molecular function	2	0.017
Binding	Molecular function	28	0.019
Catalytic activity	Molecular function	10	0.018

**Fig. 6:**
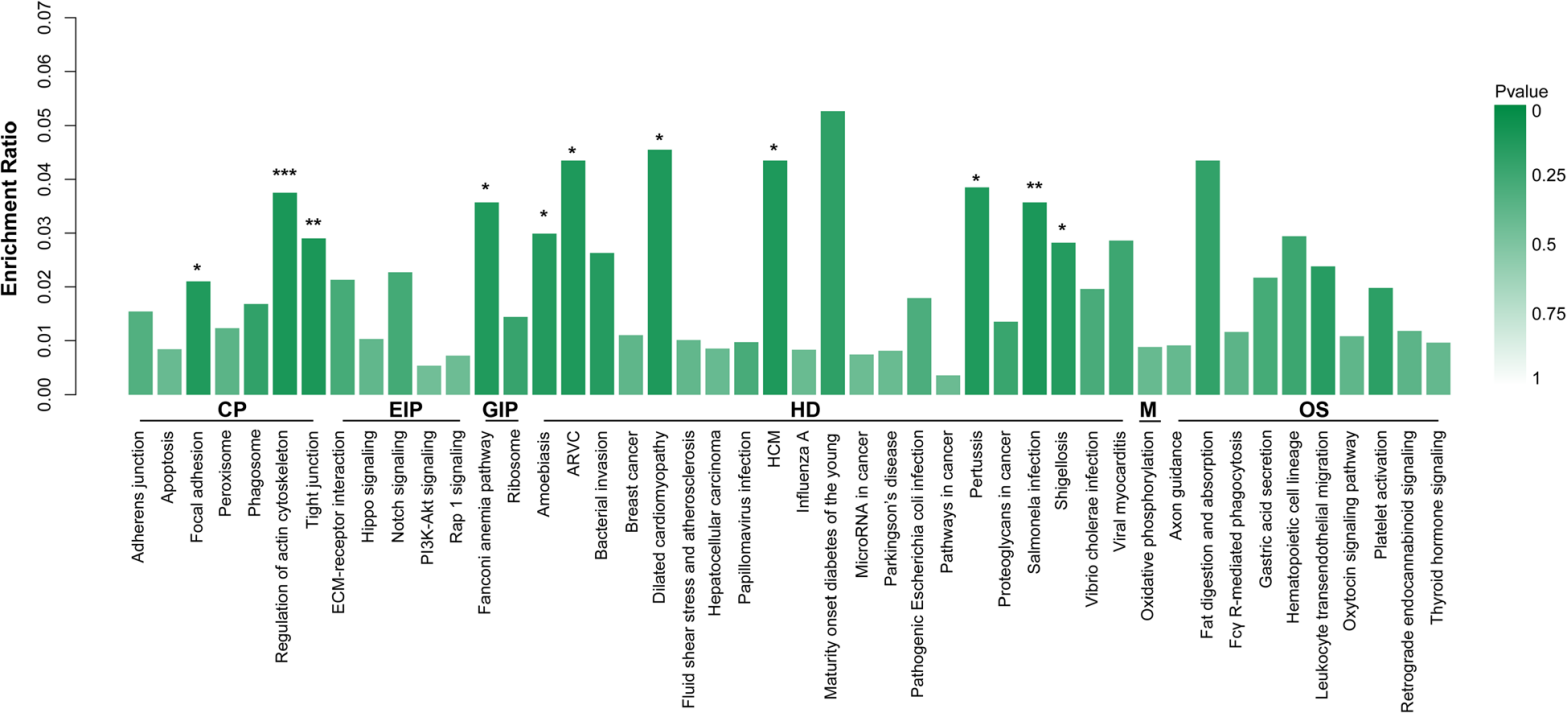
KEEG analysis. Each column is a pathway, the abscissa indicates the name and the classification, the height of the column indicates the enrichment rate, and the *p* value is represented by the color. The darker the color, the more significant. Results showed that 11 pathways were significant changed in the COM treated HK-2 cells as compared to its untreated control. ***, *p* < 0.001; **, *p* < 0.01; *, *p* < 0.05

There were 11 significant pathways identified in HK-2 cells in response to COM crystals (Fig. 6 and Table 4), among which the regulation of actin cytoskeleton pathway was the most significant (*p* = 0.0001), suggested that the regulation of the actin cytoskeleton was a critical part of cellular activities in response to COM crystals adhesion.

**Table 4:** KEGG pathway analysis of the differentially expressed proteins involved in COM-stimulated HK-2 cells

Pathway	Class	Count	***p*** value
Regulation of actin cytoskeleton	Cellular Processes	6	0.0001
Tight junction	Cellular Processes	4	0.004
Focal adhesion	Cellular Processes	3	0.0294
Salmonella infection	Human Diseases	3	0.0076
Dilated cardiomyopathy (DCM)	Human Diseases	2	0.0195
Arrhythmogenic right ventricular cardiomyopathy (ARVC)	Human Diseases	2	0.0211
Hypertrophic cardiomyopathy (HCM)	Human Diseases	2	0.0211
Pertussis	Human Diseases	2	0.0264
Amoebiasis	Human Diseases	2	0.041
Shigellosis	Human Diseases	2	0.0453
Fanconi anemia pathway	Genetic Information Processing	2	0.0301

### Biological interaction of differentially expressed proteins

**Fig. 7:**
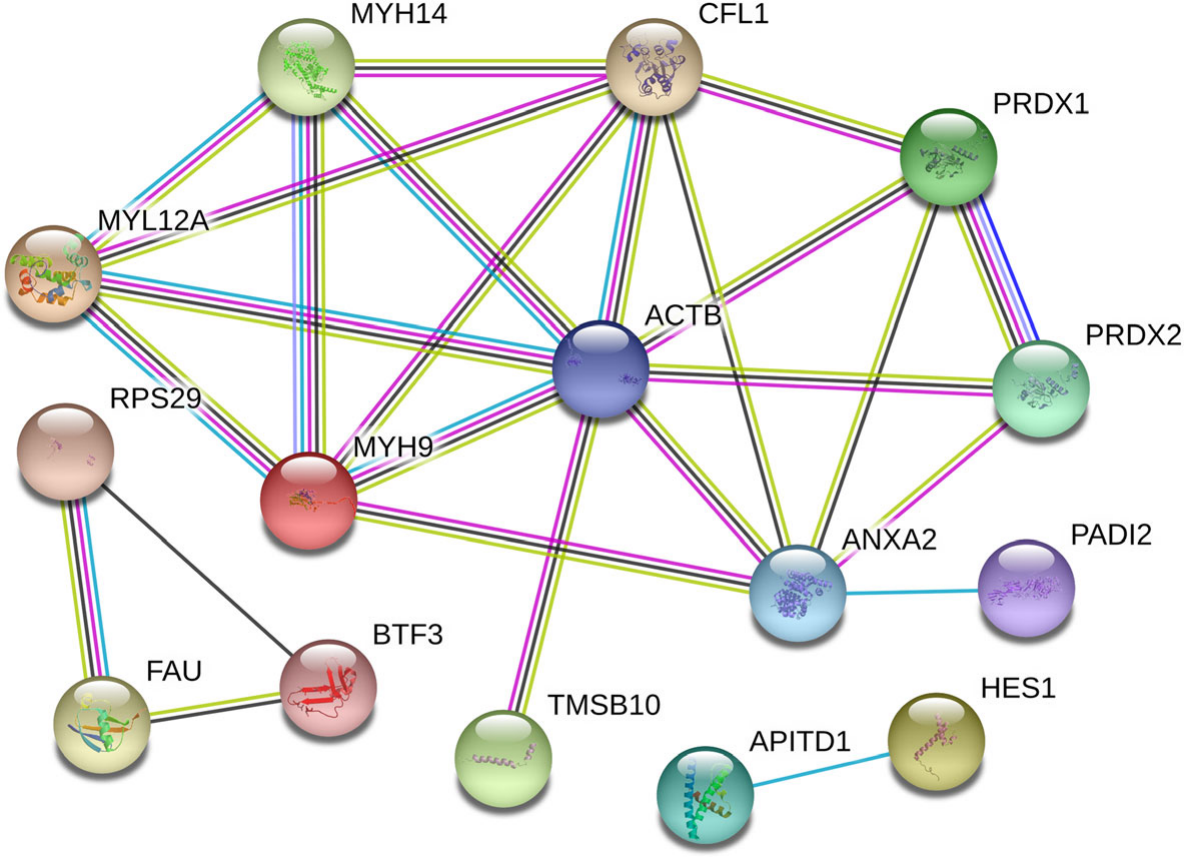
Protein interaction networks of differential expressed proteins. According to the STRING database, the protein interaction networks of the DEPs contained 8 main nodes and 21 connections. Each node in the network represents a protein molecule, and the line represents the interaction between proteins. The wider the line, the higher the score, and the narrower the line, the lower the score. The top 3 high-score hub nodes included CFL1, ACTN and MYH9. The interaction between CFL1 and ACTN exhibited the highest combined score of 0.999, suggested that may play vital roles in the adhesion of COM to the HK-2 cells

According to the information of STRING database, the protein-interaction networks of the selected DEPs contained 8 main nodes and 21 connects (Fig. 7). The top 3 high-score hub nodes included β-Actin (ACTN), Cofilin-1 (CFL1) and Myosin-9 (MYH9). Among these proteins, the interaction between CFL1 and ACTN, which were both up-regulated in COM-treated HK-2 cells, exhibited the highest combined score of 0.999. These proteins and interactions may play vital roles in HK-2 cells in response to the COM crystals adhesion.

## Discussion

Kidney stone is a common urological disease with high incidence, which remains a common health problem worldwide. At present, the mechanism of kidney stone formation is not fully understood. One of the favored theories suggests that the calcium oxalate-induced injury to renal tubular epithelial cells promotes the adherence and acumination of calcium oxalate crystals results in stone formation [27]. Given proteins are the major component of kidney stone organic matrix and considered to play a regulatory function in cell-crystal interactions and lithogenesis inside the kidney [28–31], we selected immortalized human proximal tubular epithelial cells HK-2 exposure to COM crystals to generate the cell-crystal interaction model and analyzed the altered proteomic landscape in HK-2 cells in response to COM adhesion.

In this study, we first systematically screened the DEPs profiles in COM-HK-2 model by TMT-labeled quantitative proteomics analysis. Of the 1141 identified DEPs, 699 (61.3%) proteins were up-regulated, 442 (38.7%) proteins were down-regulated. By contrast, a similar previous study performed by Chen et al. in 2010 [32] demonstrated that only 12 DEPs were identified in HK-2 cells after COM crystals stimulation. This different observation may due to the limits of methodology used by Chen et al. in their study. Here, gel-free TMT-based quantitative proteomic approach uses isobaric labels and allows for genome-wide quantitation of differences in protein expression levels [33], proved to be a more reliable and reproducible technology to analyze complex samples.

Several of proteins have been characterized to play critical roles in kidney stone formation, however, the mechanisms involved are far from clear. Tamm-Horsfall protein (THP) was identified as a kidney-specific protein, serve as a key regulator in promoting the aggregation of calcium-salt crystals and stone formation [34, 35], which is a potent immunomodulatory molecule and a disease biomarker in the urinary system [35]. Osteopontin (OPN) is another important component of calcium oxalate stone matrix [29], plays an important role in preventing the formation of calcium oxalate monohydrate (COM) kidney stones, which controls switching of calcium oxalate monohydrate morphologies in urine [36] to promote the formation of stones by promoting crystal adhesion and mediating oxidative stress and apoptosis [17, 37].

In this study, we identified that the LZTS1 (leucine zipper hypothetical tumor suppressor gene 1) protein, a full-length 596 amino acid protein with a molecular weight of 67 kD, was significantly upregulated in COM adhered HK-2 cells. Though the role and mechanism involved of LZTS1 protein was well studied in various types of malignant tumors such as stomach, lung, bladder, ovary, and kidney [38], the functional role of LZTS1 in kidney stone disease is unreported. The HES1 (hairy enhancer of split 1) protein, the core effector of NOTCH signaling pathway and considered to be a good indicator of the activation of the NOTCH signaling pathway [39, 40], exhibited significant reduce expression in COM adhered HK-2 cells. Present study is the first report to demonstrate the relation between the HES1 protein and kidney stone formation and its associated kidney injury, however, further study of the mechanism involved is in need.

Our bioinformatics analysis indicated that the GO terms of cellular process, cell part, organelles, binding and catalytic activity are significant changed in COM adhered HK-2 cells, suggested that these processes or cell structures and its related proteins are widely involved in kidney stone formation. The enriched signaling pathway identified by KEEG analysis were regulation of the actin cytoskeleton, tight junction and focal adhesion. Of these, the regulation of actin cytoskeleton showed the most significance, which plays vital roles in response to extracellular signals, spatially and temporally regulates adhesions, protrusion, contraction, and retraction [41]. Meanwhile, we analyzed the protein interaction networks of the selected DEPs and found that the CFL1, ACTN and MYH9 were the 3 high-degree hub nodes and may be involved in the pathological processes of HK-2 cells in response to the COM crystals adhesion. These findings suggested that the cell structure- and cell actin cytoskeleton dynamics had been significantly changed by COM crystals.

## Conclusion

We screened and identified key protein molecules that may be involved in the formation of calcium oxalate kidney stones, and revealed the possible signaling pathways and related disease processes, providing important potential targets and interactions for further elucidation of the pathogenesis of kidney stones. However, despite these comprehensive bioinformatics analyses, the current study has several limitations. HK-2 cells are not normal proximal tubular cells, which is a big limitation of the present study. Furthermore, the in vitro COM concentration could not perfectly mimic the status of crystals in kidney, the dose-dependent and time-dependent effects of COM stimulation were not involved. In our future investigations, we hope to perform additional proteomics analyses on normal proximal tubular cells/animal models of different time points and experimental validation to enrich this study.

## Data Availability

The datasets used during the current study are available from the corresponding author on reasonable request.

## Abbreviations

COM: Calcium Oxalate Monohydrate
DEP: Differential expression protein
TMT: Tandem Mass Tag
KEEG: Kyoto Encyclopedia of Genes and Genomes
GO: Gene Ontology
CC: Cellular Component
BP: Biological Processes
MF: Molecular Function
OS: Organismal Systems
HD: Human Diseases
CP: Cellular Processes
EIP: Environmental Information Processing
GIP: Genetic Information Processing
M: Metabolism
